# Decision-making dynamics are predicted by arousal and uninstructed movements

**DOI:** 10.1016/j.celrep.2024.113709

**Published:** 2024-01-26

**Authors:** Daniel Hulsey, Kevin Zumwalt, Luca Mazzucato, David A. McCormick, Santiago Jaramillo

**Affiliations:** 1 Institute of Neuroscience, University of Oregon, Eugene, OR 97405, USA; 2 Department of Biology, University of Oregon, Eugene, OR 97405, USA; 3 Departments of Physics and Mathematics, University of Oregon, Eugene, OR 97405, USA; 4 Senior author; 5 Lead contact

## Abstract

During sensory-guided behavior, an animal’s decision-making dynamics unfold through sequences of distinct performance states, even while stimulus-reward contingencies remain static. Little is known about the factors that underlie these changes in task performance. We hypothesize that these decision-making dynamics can be predicted by externally observable measures, such as uninstructed movements and changes in arousal. Here, using computational modeling of visual and auditory task performance data from mice, we uncovered lawful relationships between transitions in strategic task performance states and an animal’s arousal and uninstructed movements. Using hidden Markov models applied to behavioral choices during sensory discrimination tasks, we find that animals fluctuate between minutes-long optimal, sub-optimal, and disengaged performance states. Optimal state epochs are predicted by intermediate levels, and reduced variability, of pupil diameter and movement. Our results demonstrate that externally observable uninstructed behaviors can predict optimal performance states and suggest that mice regulate their arousal during optimal performance.

## In brief

Performance during auditory and visual discrimination tasks fluctuates, often with abrupt shifts in stimulus-response relationships. Hulsey et al. demonstrate that these fluctuations coincide with changes in pupil diameter and movement levels, with optimal performance occurring during moderate, stable arousal. Their results suggest differential regulation of arousal across task-engagement states.

## Graphical abstract

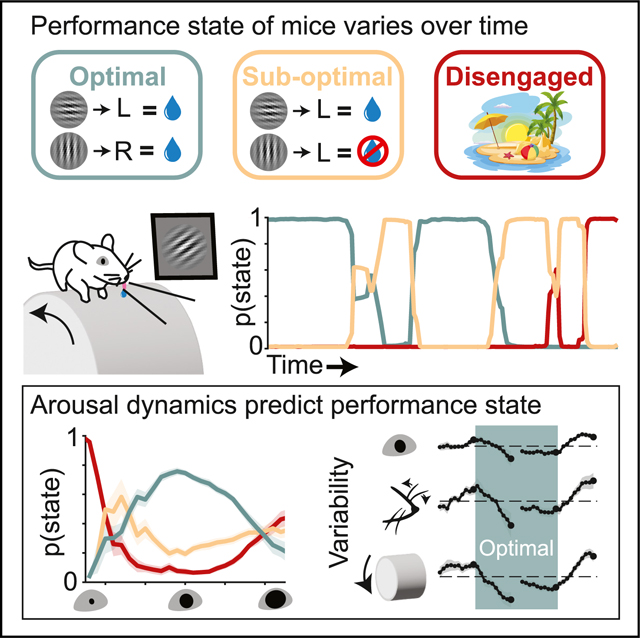

## INTRODUCTION

Behavioral and neural responses are notoriously variable across task trials in animals, including humans.^1^ Recent studies have demonstrated that this variability is not random but rather arises from changes in neural and physiological states.^1–3^ For example, arousal levels fluctuate on a moment-to-moment basis, and a significant fraction of neural and behavioral variability can be predicted by pupil diameter, which is tightly linked to arousal.^4^ Uninstructed movements also influence neural dynamics, as shown in the visual system^5–9^ and reported ubiquitously across the cerebral cortex.^10–12^ Methods for studying behavioral variations in relation to arousal vary but can lead to cohesive views of the nervous system and its function.^13^ For instance, the Yerkes-Dodson law, first proposed in 1908 and developed and modified through the years since, outlines an inverted-U relationship between arousal and performance on difficult tasks, with optimal performance occurring at intermediate arousal.^14^

While some studies have successfully demonstrated a lawful inverted-U-shaped relationship between behavioral performance and arousal, others have failed to achieve similar findings.^4,15^ Uncovering the mechanisms of behavior is highly dependent upon detecting and understanding these behavioral- and neural-state-dependent processes. One possible reason for this inter-study variability is that certain sub-states of behavior were undetected or non-occurring. Further, these prior studies on the impact of arousal and movement consider moment-to-moment fluctuations but do not address structured variations across time during task performance. Therefore, developing a robust strategy for detecting variations in behavioral strategy, and understanding how they interact, is an essential prerequisite to revealing the underlying neural mechanisms of behavior. A recent study^16^ showed that mice and humans alike express a handful of discrete strategies during decision-making tasks and switch between them within the same experimental session. These results advance classical models of performance by accounting for structured fluctuations in engagement strategies. However, the precise relationship between arousal, movements, and discrete strategic performance states is not known.

Here, we address this fundamental relationship by linking transitions between decision-making strategies to changes in arousal (as measured by pupil diameter) and uninstructed movements (as measured by the motion energy of a video of the face [face motion energy] and locomotion speed). We identified epochs of optimal task performance and showed that trained animals maintain them for longer durations when compared to periods of sub-optimal strategic engagement. Consistent with the Yerkes-Dodson law, we found a striking inverted-U relationship between the likelihood of optimal state occupancy and both pupil diameter and uninstructed movement in auditory and visual discrimination tasks. Similarly, we found a U-relationship between the probability of task disengagement and pupil diameter/uninstructed movement. Furthermore, reduced variability in pupil diameter and movement measures signaled transitions into the optimal state and could be used to predict the onset and offset of decision-making states. Our results reveal that a significant fraction of behavioral variability can be accounted for by modeling variations in sustained behavioral state/strategy, which can be predicted by shifts in a subject’s arousal and uninstructed movements, and further suggest that controlled arousal is key to maintaining optimal performance.

## RESULTS

### Mice switch between several performance states during auditory and visual decision-making

**Figure 1.**
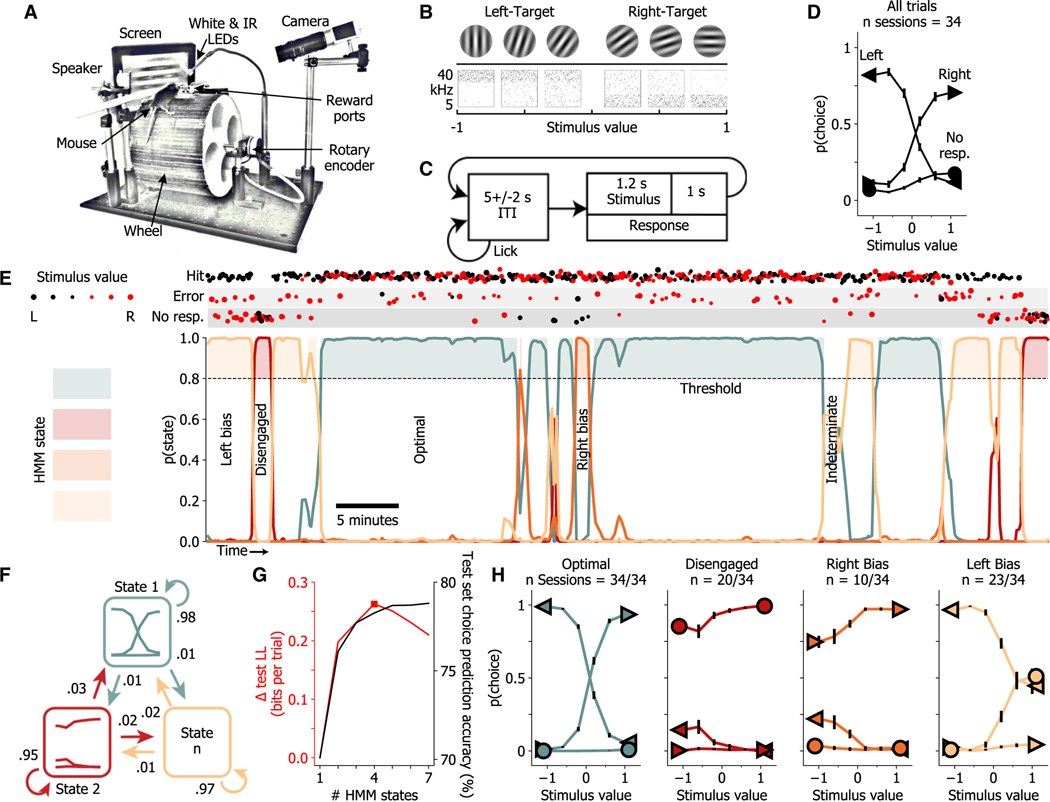
GLM-HMM reveals epochs of optimal performance amid periods of sub-optimal strategies and complete disengagement (A) During task performance, mice were head fixed above a running wheel in front of a screen and speaker. (B) Mice were trained to categorize the angle of a drifting Gabor patch as mostly vertical vs. horizontal or the concentration of pure tones in a tone cloud as mostly high vs. low frequency. Negative stimulus values are associated with left-target stimuli and positive values with right-target stimuli. (C) Stimuli were presented after a random duration (5 ± 2 s) of licking quiescence. Subsequently licking the corresponding (L/R) reward port resulted in water reward delivery. See also Figure S1. (D) Performance of an example mouse during the visual task, presented as the probability of each choice type (left [<], right [>], no response [o]) given each possible stimulus. (E, top) Structured fluctuations appear in trial outcomes during example session. Stimulus values for each trial are represented by marker size and color (size increases with ease of discrimination). (E, bottom) GLM-HMM state posterior probabilities capture fluctuations in performance dynamics. A confidence criterion of 80% was set for inclusion of a trial in a state for further analyses. (F) GLM-HMM, with GLMs of psychometric performance for each hidden Markov state, and state-transition probabilities. (G) An appropriate number of states, denoted here by a red square, was determined using cross-validated test-set log likelihood. Cross-validated choice prediction accuracy continues to increase with number of states. Log-likelihood values are plotted as change in relation to a one-state model. See also Figure S2 for all individual subjects. (H) Performance of example mouse during trials sorted by GLM-HMM states. Some states occurred in only a sub-set of sessions (indicated by n sessions). Data represent the mean ± SEM across sessions.

To identify performance states/strategies explored by an animal during perceptual decision-making, we trained mice for extended sessions on either an auditory or a visual stimulus discrimination task (Figures 1A–1C). Both versions of the task required mice to lick a left or right reward port to categorize the stimulus. For the auditory discrimination task, tone clouds were differentiated by the concentration of frequencies in high- or low-frequency bands, while in the visual task, the angle of a drifting Gabor patch was categorized as closer to a vertical or horizontal orientation (Figure 1B). We specifically sought to explore a broad range of arousal in our mice, from drowsy to highly aroused. Two factors of the task design were chosen specifically to promote this broad range of arousal during performance. First, mice were trained on a running wheel to allow high levels of arousal, such as those that occur with sustained running. Second, long inter-trial intervals (5 ± 2 s, which reset upon licking; Figure 1C) were used to prevent arousal responses of each trial from influencing subsequent trials (Figure S1), which allows a reduction of arousal between stimuli, facilitating access to lower arousal levels such as those occurring during prolonged quiescence. Mice had three options available in each trial: to lick left, lick right, or not respond. Indeed, we found that subjects sometimes did not respond following a stimulus, so we considered every stimulus presentation during analysis, including trials with no response (Figure 1D).

Examining the behavior during single sessions revealed that mice switch rapidly between epochs of varying stimulus-response contingencies (Figure 1E, top). During the first 5 min, the subject shown in this figure responded predominately to both left- and right-target stimuli by licking to the left−thus exhibiting a strong left response bias. After several (30) trials of this left-bias behavior, the animal abruptly stopped responding to either left- or right-target stimuli and therefore was disengaged from the task (Figure 1E, top). This 14-trial-long period of disengagement was followed by another period of left bias that lasted 29 trials. Suddenly, the animal started responding accurately to both left- and right-target stimuli, in what we term the optimal state. The subject remained in this state for a prolonged period of time (~20 min). The remainder of this behavioral session could be roughly described as transitions between states characterized by either left-bias, right-bias, disengaged, optimal, or intermediate (indeterminate) states (Figure 1E, top).

To more accurately and automatically capture these dynamics in performance, we used hidden Markov models with generalized linear model emissions (GLM-HMMs; Figure 1F). Each HMM state (further referred to as a strategic or performance state) corresponds to a psychometric curve, captured by a GLM. GLM-HMMs have been previously used on two-alternative forced-choice tasks with only two possible outcomes (L and R choices), leading to Bernoulli emissions.^16^ Here, we extended this model to include multinomial emissions representing the three possible choices (L, R, no response). This proved crucial to capturing the full range of performance states, including disengagement. The model parameters include a transition probability matrix capturing the transition rates between different states, as well as GLM observation parameters representing the stimulus weights and bias of the psychometric curve in each state (Figure 1F). The number of performance states for the model was determined for each mouse using cross-validation. For the example mouse shown in Figure 1G, this method yielded 4 discrete states. A final model was fit to all available data for a subject. The models yield posterior probabilities for each decision-making state in each trial, revealing long-lived states detected with high confidence and lasting for tens of consecutive trials (Figure 1E, bottom). Most transitions between states were abrupt, but some were slower, resulting in periods with low posterior probabilities across HMM states. In order to assess the prevalence of such indeterminate periods and study truly distinct states, we set a minimum posterior probability threshold of 0.8 for inclusion of a trial within a discrete state.

Psychometrics generated from trials of each performance state yielded clearly interpretable and distinct task-engagement profiles. For the representative subject presented in Figure 1, decision-making performance alternates between a state containing optimal performance, where responses were lawfully guided by the sensory stimulus; a disengaged state, with no response following stimuli; and sub-optimal left- and right-bias states, where the subject responded predominately in one direction regardless of the stimulus identity (Figure 1H). From here on, we refer to the state in which the animal is responsive and the resulting psychometric curves indicate that responses are correctly guided by the stimulus, without significant leftward or rightward bias, as the optimal state (see Figure 1H).

To compare the accuracy of this GLM-HMM technique to a model that assumes an animal does not switch between performance states, we use each model to predict the choices of the mouse on each trial. We again used cross-validation and generated models trained on 80% of trials from each session, with-holding blocks of 20% of trials as a test set. The state of each test-set trial was inferred using the posterior probability of state occupancy of the preceding trials and the state transition matrix, and a choice was predicted using the GLM for the inferred state. A one-state GLM-HMM, equivalent to assuming stationary performance during training, correctly predicted the choice of the example mouse on 70% of trials, while the best-fit 4-state GLM-HMM correctly predicted 78% of choices (Figure 1G).

**Figure 2.**
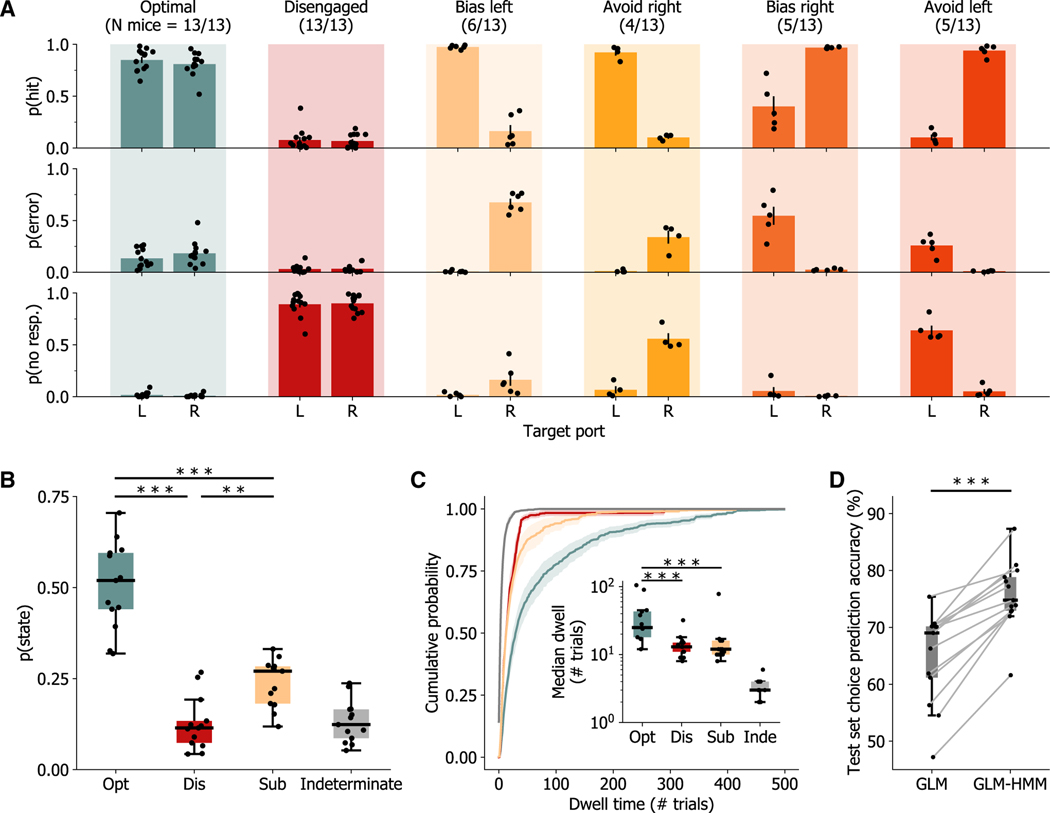
GLM-HMMs converge on six stereotypical performance states across mice (A) Probability of hit, error, or no response for each mouse in each performance state. Each mouse had an optimal (L/R choice guided by stimulus) and disengaged (non-responsive to stimuli) state, along with one or more sub-optimal performance states. Sub-optimal states could be classified into four categories−bias left or right, where the mouse responds to the opposite stimulus with an error, and avoid left or right, where a stimulus of one direction was not responded to, while the other was responded to with a lick to the correct side. Data are represented as mean ± SEM across states in available subjects. (B) Mice were in the optimal state in around half of all trials. (C) Cumulative probability of performance state dwell times, averaged across individual mice. Inset: upon each entry into an optimal state, mice remained in the optimal state for longer periods compared to other performance states. (D) Accounting for strategic shifts in behavior with GLM-HMMs significantly increases model choice prediction accuracy. In (B)–(D), the box extends between the lower and upper quartiles, with a line at the median, and whiskers extend to the last data point within 1.5 times the interquartile range. N = 13 mice. Statistics are only shown for discrete state comparisons in (B) and (C). **p < 0.01 and ***p < 0.001 using Wilcoxon signed-rank tests in (B) and (C) and rank-sum tests in (D).

To examine the generality and robustness of these observations, we fit GLM-HMMs separately to each of the 13 mice that reached proficiency on the sensory discrimination task (8 auditory and 5 visual task performers). While there was diversity in the best number of states across subjects (3–5 states; Figure S2), six stereotypical performance states emerged as follows. Each subject exhibited both optimal (state 1) and disengaged (state 2) states, along with at least one additional sub-optimal performance state. In sub-optimal states, subjects either responded predominantly to the left (or right) regardless of stimulus value (yielding states 3 and 4, left or right biased) or responded correctly to left-target (right-target) stimuli while with-holding responses to right-target (left-target) stimuli (yielding states 5 and 6, avoid right or avoid left; Figure 2A). Subjects spent the largest proportion of trials in the optimal performance state (Figure 2B; optimal state (opt.) vs. disengaged state (dis.) p = 2.4 × 10^−4^, opt. vs. sub-optimal state (sub.) p = 2.4 × 10^−4^, opt. vs. indeterminate state (inde.) p = 2.4 × 10^−4^, Wilcoxon signed-rank tests), and only 13.1% ± 1.6% of trials were in an indeterminate state. Additionally, epochs of the optimal state lasted longer than those of sub-optimal states (Figure 2C; opt. vs. dis. p = 2.4 × 10^−4^, opt. vs. sub. p = 2.4 × 10^−4^, Wilcoxon signed-rank tests), and the median transition time between discrete states (dwell time in indeterminate states) was 3.4 ± 0.3 trials. Finally, we determined the choice prediction accuracy of the models for each mouse and found that the best-fit GLM-HMM correctly predicted 75.7% ± 1.7% of choices, a significant 10.8% ± 1.3% improvement over the classical static-performance assumption (Figure 2D; p = 2.4 × 10^−4^, Wilcoxon signed-rank tests). These observations demonstrate that multinomial GLM-HMMs describe task performance better than classical static-performance models and that these models can be used to identify epochs of optimal performance. These models also revealed that trained mice selectively maintain optimal strategic states longer than sub-optimal strategies during task performance.

### Arousal and uninstructed movement are regulated during optimal performance

**Figure 3.**
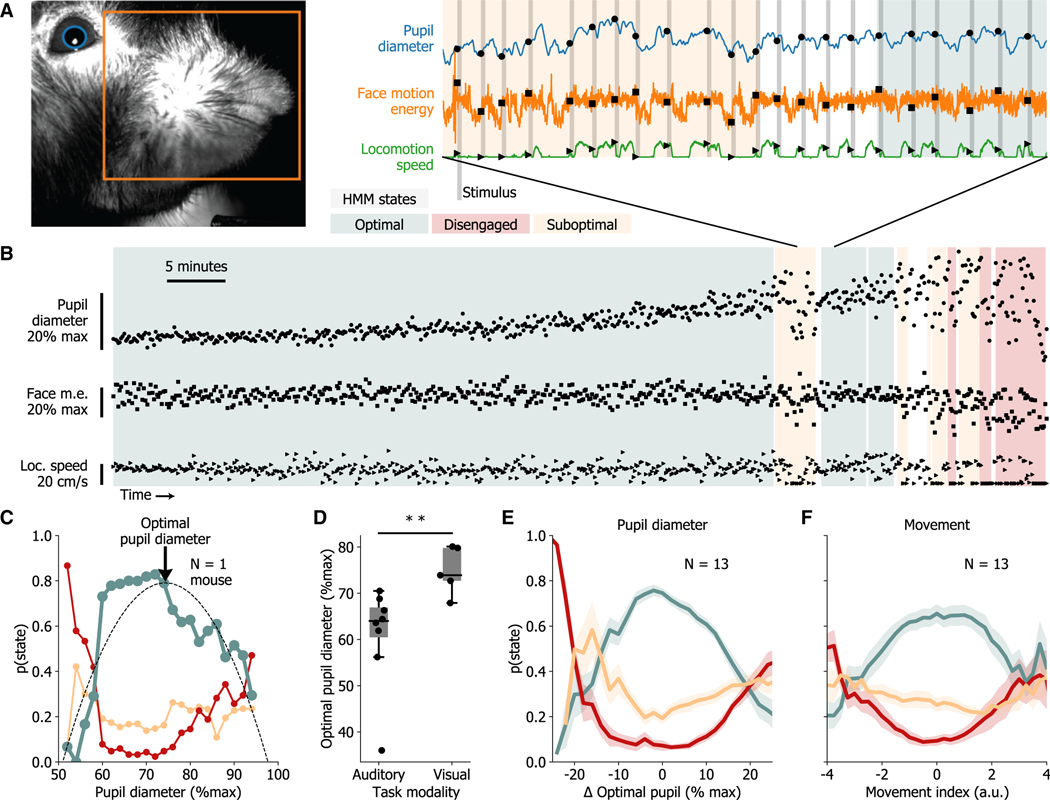
Optimal performance state is characterized by intermediate levels of both pupil diameter and uninstructed movement (A) Pupil diameter, face motion energy, and locomotion speed before stimulus presentation are used for further analysis. (B) Example session from a mouse performing the visual task shows shifts in arousal measures that coincide with GLM-HMM state changes. Note restricted variance and range of measures in optimal state as compared to disengaged or sub-optimal states. (C) Probability of GLM-HMM states across pupil diameters for an example mouse. Optimal state probability is fit with a polynomial function to estimate optimal pupil diameter for each mouse. See Figure S3 for all individual subjects. (D) Optimal pupil diameter is significantly larger in mice performing the visual task (N = 5) when compared to those performing the auditory task (N = 8). (E) Average probability of GLM-HMM states across all mice as a function of distance from optimal pupil reveals a robust inverted-U relationship with optimal state occupancy. (F) A movement index, combining face motion energy and locomotion speed, also has an inverted-U relationship with optimal state probability. There is a marked U-shaped relationship between both pupil diameter and movement index and the probability of being in the disengaged state. See also Figures S4 and S5 for analysis of individual movement measures and movement index for individual subjects. (D) **p < 0.01 using Wilcoxon rank-sum test. Plots in (E) and (F) represent the mean ± SEM across mice (N = 13).

How do arousal and uninstructed movement relate to strategic states and their transitions? We hypothesized that there would be lawful relationships between discrete strategic performance states and both arousal and movement measures. To examine this idea, we monitored measures of pupil diameter, face motion energy, and locomotion speed of mice during task performance. Our analyses used the value of each measure at the time point sampled immediately before the onset of a stimulus to determine their influence on variations in the subject’s response (Figure 3A). A comparison between the time course of these arousal measure values and the HMM state dynamics within single sessions revealed precise relationships between these variables, providing support for our hypothesis (Figure 3B). To quantify these relationships, we examined how differences in pupil diameter and movements measures (i.e., face motion energy and locomotion speed) affected the probability of state occupancy (e.g., probability of being in the optimal state). Interestingly, we found a striking inverted-U relationship between the probability of optimal state occupancy and pupil diameter, where the likelihood of being in the optimal state is highest at intermediate pupil diameters (Figure 3C). The inverted-U relationship was found in nearly all subjects, and the pupil diameter that gave the highest probability for being in the optimal state was calculated for each (Figure S3). Subjects performing the auditory task had significantly smaller optimal pupil sizes than those performing the visual task (even though illumination conditions were the same during both tasks), suggesting that optimal arousal levels may be task-modality dependent (Figure 3D; p = 0.008, Wilcoxon rank-sum test).

To visualize the relationship between pupil diameter and performance states across mice, we normalized the x axis (pupil diameter) to the “difference from the optimal pupil diameter” for each subject to account for individual differences in the optimal pupil diameter (Figure 3E). Similar to the inverted-U relationship between pupil diameter and the probability of being in the optimal state, we found a prominent U relationship between the probability of being in the disengaged state and pupil diameter. At either low or high pupil diameters, there was a dramatic increase in the probability of mice being in the disengaged state and a consequent large decrease in the probability of the animal being in the optimal state (Figure 3E). These relationships were maintained when considering only trials where the mice were stationary. Similar analysis of individual movement measures reveals more heterogeneous relationships across subjects. While some subjects’ face motion energy forms an inverted-U relationship with optimal state occupancy, many mice have a monotonically increasing relationship (Figure S4). Similarly, locomotion speed forms an inverted-U relationship for some mice but has a flat or monotonically decreasing relationship in others (Figure S4). These observations are reflected by significantly reduced second-order cross-validated regression fits of the probability of optimal state occupancy against face motion energy and locomotion speed when compared to pupil diameter (pupil diameter: r^2^ = 0.72 ± 0.03; face motion energy: r^2^ = 0.49 ± 0.08, Wilcoxon signed-rank test vs. pupil: p = 0.04, locomotion speed: r^2^ = 0.44 ± 0.07, Wilcoxon signed-rank test vs. pupil: p = 0.002).

Locomotion and facial movements are inter-related but convey information at differing magnitudes of movement (i.e., face motion energy measurements are sensitive to small movements but lose fidelity during locomotion when they are ubiquitous, while locomotion speed provides a continuous measure of high-magnitude body movements but no information while a subject is stationary). Owing to this, we combined these two measures into a single continuous measure of uninstructed movement levels termed the “movement index,” which is equally correlated with each measure (see STAR Methods and Figure S5). Using this combined movement index, a clear inverted-U pattern emerged, showing that optimal performance state occupancy peaked at intermediate levels of uninstructed movement, similar to that of pupil diameter (regression of movement index vs. optimal state, cross-validated r^2^ = 0.63 ± 0.06; Wilcoxon signed-rank test vs. pupil diameter: p = 0.34; Figures 3F and S5). These correlations translate to a significant decrease in the absolute difference from the calculated optimal pupil diameter during optimal state occupancy as compared to sub-optimal or disengaged states (optimal: 7% ± 0.3%, disengaged: 12.6% ± 1%, sub-optimal: 9% ± 0.5%; opt. vs. dis. p = 2.4 × 10^−4^, opt. vs. sub. p = 4.8 × 10^−4^, Wilcoxon signed-rank tests) and a trend toward larger raw face motion energy during optimal state occupancy (optimal: 0.48 ± 0.02 a.u., disengaged: 0.43 ± 0.3 a.u., sub-optimal: 0.44 ± 0.03 a.u.; opt. vs. dis. p = 0.06, opt. vs. sub. p = 0.04, Wilcoxon signed-rank tests) but no significant differences in raw locomotion speed (optimal: 0.14 ± 0.02 m/s, disengaged: 0.14 ± 0.2 m/s, suboptimal: 0.14 ± 0.02 m/s; opt. vs. dis. p = 0.84, opt. vs. sub. p = 1, Wilcoxon signed-rank tests).

**Figure 4.**
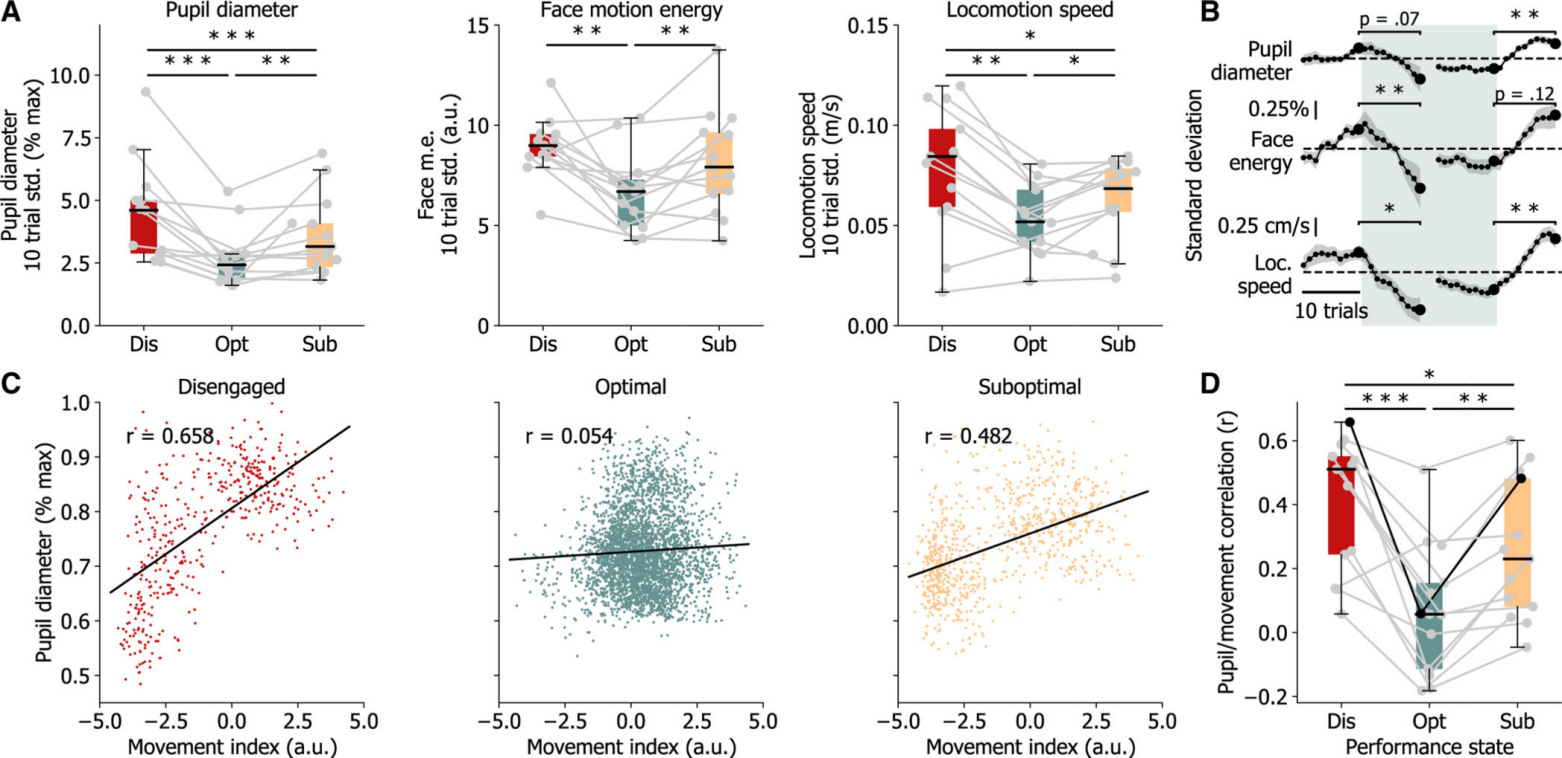
Pupil diameter and movement measures have lower trial-to-trial variability and are decoupled during optimal performance (A) Variability across the past ten trials for all recorded measures is reduced during optimal state occupancy. (B) Variability of all recorded measures shift concurrent with state transitions, decreasing upon entering the optimal state and increasing when exiting the optimal state. Plotted data represent the change in the standard deviation of each measure across the previous ten trials for trials surrounding optimal state transitions. Data were averaged across state transitions for each subject. Dashed line represents no change. (C) Data from an example subject showing movement and pupil diameter are correlated while disengaged from the task but are decoupled during optimal engagement. See also Figure S6 for correlations with individual movement measures. (D) Pupil-movement correlations are significantly lower in optimal state across mice. In (A) and (D), boxes extend between the lower and upper quartiles, with a line at the median, and whiskers extend to the last data point within 1.5 times the interquartile range. *p < 0.05, **p < 0.01, and ***p < 0.001 using Wilcoxon signed-rank tests. (B) plots represent the mean ± SEM across mice (N = 13). Lines in (C) represent linear regression fits, and r values are Pearson correlation coefficients.

We further hypothesized that performance states would differ not only in the values of pupil and movement measures but also in the variability of these values over the past ten trials. For all arousal measures recorded−pupil diameter, face motion energy, and locomotion speed−we found significantly reduced variability during optimal performance state occupancy as compared to sub-optimal or disengaged states (Figure 4A). Strikingly, this decrease in variability predicts transitions into and out of the optimal state (Figures 3B and 4B). Pupil diameter generally follows fluctuations in movement (Figure S1), but their relationship changes during task performance. During optimal state epochs, the correlation between pupil diameter and the movement index is significantly reduced across subjects (Figures 4C and 4D). This decrease in correlation is also seen when considering individual measures of face motion energy and locomotion speed (Figure S6). These results suggest that arousal levels may be differentially regulated during task engagement to maintain optimal levels for performance.

### Arousal measures predict optimal performance state

**Figure 5.**
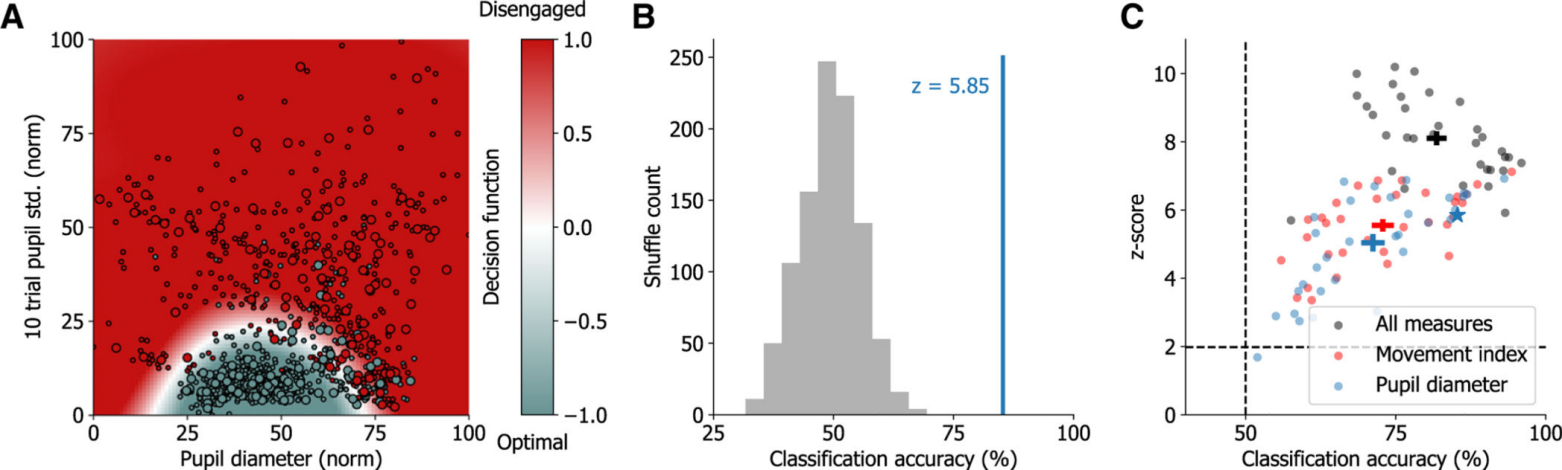
Pupil diameter and movement index predict optimal state occupancy (A) Example decision function of a support vector machine (SVM) classifier with a radial basis function kernel using pupil measures of diameter and trial-to-trial variability to predict performance states. One of five cross-validated folds is shown. Color bar represents decision function confidence, with white representing the decision boundary. Small circles: training set; large circles: test set. (B) Example of *Z* scoring test set accuracy of performance state decoder trained on empirical data (blue line; 1-fold from A, *Z* scored against classifiers trained with state-identity-shuffled data [gray distribution]). (C) SVM classifications were made for each available state against the optimal state per mouse (i.e., disengaged vs. optimal, bias left vs. optimal, etc.). Data points represent average performance state classification accuracy across test sets and *Z* scores against test-set accuracy of classifiers trained on state-shuffled data. The blue star represents the classification from (A) and (B). Shuffling all features except movement index or pupil variables (both value and 10-trial standard deviation) reveals the individual contribution of each measure to state classification. Crosshairs represent mean ± SEM across all state classifications (n = 32; 13 optimal vs. disengaged, 19 optimal vs. sub-optimal). See also Figures S7 and S8 for further analysis of individual measure contributions.

To quantify the extent to which different performance states can be predicted based on the recorded arousal and movement measures, we implemented a cross-validated state classification analysis. For each subject, we trained a classifier to discriminate between optimal performance state and disengaged or sub-optimal states using the value and variability of each measure as features. Figure 5A shows an example of such a classifier using the value and trial-to-trial variability of pupil diameter. We found that for all subjects, performance states could be significantly predicted using the recorded arousal measures with high accuracy (opt. vs. dis. = 88.2% ± 1.8%; opt. vs. sub. = 77.7% ± 2%; Figures 5B and 5C; statistical significance was assessed by comparing empirical classification accuracy to surrogate datasets obtained by shuffling class labels in the training set). To assess the contributions of individual arousal measures to performance state decoding, sub-sets of measures were shuffled in the training sets of the classifiers. Pupil diameter (value and trial-to-trial variability) was the best individual raw measure for state classification (Figure S7; pupil vs. face motion energy p = 3.9 × 10^−3^, pupil vs. locomotion p = 0.106, Wilcoxon signed-rank tests over optimal state classifications). Interestingly, we found that the best predictor of performance state was the computed movement index incorporating face motion energy and locomotion speed, which encoded more information about states compared to pupil diameter (Figure 5C; pupil vs. movement index p = 0.024, Wilcoxon signed-rank tests over optimal state classifications). To understand the contribution of individual measures, we visualized classifier decision functions using individual measures (Figures 5A and S7C), which were consistent with the state probability relationships for individual measures (Figures 3E, 3F, and S4).

To further quantify the unique relationships between each measure and performance state probabilities, we fit linear regressions to state probabilities for each mouse using features of the individual measures (pupil, face motion energy, and locomotion speed values per trial), along with their ten-trial standard deviations, second-order polynomials, and interaction terms. Consistent with other analyses, weights for the quadratic terms fit to predict optimal state probability were negative for pupil diameter across mice, indicating an inverted-U-shape relationship, and the weights were positive when fit to disengaged state probabilities, indicating a U-shaped relationship (Figure S8A). This was also true for quadratic terms of locomotion speed, while face motion energy had weights centered around zero across subjects. Consistent with previous analyses, the weights for the standard deviations of pupil diameter and locomotion speed across trials were consistently negative when fit to optimal state probability and positive when fit to the disengaged state; however, weights were centered around zero for variability of face motion energy. We also fit regressions using the combined movement index instead of individual movement measures. The movement index had the most consistent weights across mice, also exhibiting inverted-U dynamics in relation to optimal state probability, with negative weights for the second-order polynomial term (Figure S8B). Interestingly, the only consistently weighted feature across fits to sub-optimal state probabilities was the interaction between pupil diameter and the movement index (Figure S8B; 0.42 ± 0.11), which was also significantly different than weights fit to optimal state probability (−0.95 ± 0.21; p = 3.4 × 10^−6^, Wilcoxon rank-sum test).

## DISCUSSION

In this study, we provide evidence that a significant fraction of behavioral variability arises from rapid transitions between identifiable substates, that pupil diameter and uninstructed movements can accurately predict these substates and their transitions (including epochs of optimal performance), and that controlled arousal is key to maintaining optimal performance. First, using GLM-HMM modeling of task performance, we show that well-trained mice alternate between discrete strategic performance states and that accounting for these states significantly improves the accuracy of behavioral modeling. Our methods, including trials where the subject does not respond, allowed us to go beyond previous reports^16^ and identify disengaged and other low performance states in addition to optimal performance states. Interestingly, we found that mice selectively maintained optimal states for longer durations than sub-optimal and disengaged states (Figures 1D and 2C). Second, consistent with the classical Yerkes-Dodson law, we demonstrate that optimal task performance occurs at intermediate arousal levels during sensory discrimination tasks (Figures 3E and S3). While previous reports demonstrated this relationship during auditory detection,^4^ it has not been reported during visual tasks.^15^ Here, we find an inverted-U relationship during both auditory and visual discrimination task performance. There was a significant difference in optimal pupil diameters between sensory modalities, suggesting modality-specific modulation of arousal during optimal performance. Third, we found significantly lower trial-to-trial variability in both pupil diameter and movement measures during optimal performance, with rapid shifts in variability at transitions into and out of the optimal state (Figures 3B and 4B). Finally, we found that arousal measures, taken prior to stimulus presentation, can be used to accurately predict the discrete task performance states produced by GLM-HMM modeling (Figure 5C). By using GLM-HMMs, we extend the analysis of arousal measures beyond average characterizations of a moment-to-moment performance-arousal relationship in mice and suggest strategic regulation of arousal levels during optimal performance.

Here, we used recently implemented GLM-HMMs^16,17^ and advanced their utility to account for stimuli not responded to by the subject. This extended model accounts for additional behavioral states present during decision-making in mice, in particular revealing states of disengagement and selective avoidance of a particular stimulus category (i.e., left or right target). Using an individual model for each subject accounted for differences in individual psychometric response patterns and state-transition probabilities, and cross-validated model selection prevented overfitting of models. Despite individual differences, when comparing across subjects, a consistent view of task performance emerged, with a limited number of easily classifiable, congruent strategies in the population. With the trial-wise state probability confidence threshold of 0.8 used, across mice, over 85% of trials were classified in a discrete state, and the median transition time between states was under 4 trials, significantly shorter than the average dwell times of 37 trials in the optimal state and of ~15 in disengaged and sub-optimal states. While this does not exclude the possibility of continuous dynamics during decision-making behavior, GLM-HMMs provide a powerful tool for studying distinct task performance states. In the current study, we observed arousal-related differences between optimal and sub-optimal strategic states, but different performance states may additionally have markedly different neural activity and/or functional connectivity correlates. Studying neural data regarding decision-making could benefit from comparing not only hits and errors but also differences between similar trial outcomes during various strategic performance states.

Arousal and movement can be indexed by numerous, often correlated measures.^1^ Here, we use simple and accessible measures of pupil diameter, face motion energy, and locomotion speed. Head-fixed mice on a running wheel can exhibit a broad range of arousal and movement, from sleep to rapid locomotion. To promote a broad range of arousal in our mice, we both allowed mice to locomote rapidly on a running wheel during task performance (therefore allowing access to higher levels of arousal and uninstructed movement) and used long inter-trial intervals to promote periods of behavioral quiescence and task disengagement. Preliminary results indicated that short inter-trial intervals typically resulted in a lack of lower levels of arousal (e.g., smaller pupil diameter during behavioral quiescence) and biased our results toward intermediate to high arousal levels. Trials were also delivered at random intervals and were not self-initiated, preventing constraints on the subject’s state before a trial. These factors were crucial for observations of an inverted-U relationship between optimal state probability and pupil diameter (Figure 3E).

We additionally saw an inverted-U relationship between a movement index combining face motion energy and locomotion speed with optimal state probability, similar to that of pupil diameter (Figure 3F). The measurement of face motion energy individually showed a monotonically increasing relationship with optimal performance state occupancy. Onset of facial movements tracks rapid shifts in intracellular^6,18,19^ and neuromodulatory neural dynamics^19–21^ but lacks fidelity during prolonged bouts of movement and locomotion, when face movement is ubiquitous. Conversely, locomotion speed does not capture the onset of whisking and movement but provides a continuous measure of high-energy movements. Thus, our face motion energy measure is well suited for detecting both lower levels of uninstructed movements (e.g., movements that do not include the large skeletal muscles of the limbs and body), while locomotion speed is better suited for detecting higher levels of these movements. While some mice had an inverted-U relationship between these individual measures and optimal performance state, combining the two movement measures allowed for one index to account for a wide range of movement levels and resulted in an inverted-U relationship with optimal performance state across all mice. Based on these factors, we consider pupil diameter to be the best easily accessible, individual measure of arousal. While complex analysis of video data could lead to more precise measures of unique movement profiles for individual subjects, the simple movement index used here is sufficient to make accurate predictions of task performance state and corroborates a long-standing physiological phenomenon first described by Yerkes and Dodson.^14^

What factors contribute to the inverted-U phenomenon during task performance? In the auditory cortex, membrane potential dynamics at intermediate arousal levels are ideal for stimulus detection.^4^ Task engagement also influences auditory processing in multiple brain regions, and changes are congruent and often overlapping with pupil-linked effects.^22^ In this case, maintaining an intermediate arousal level could directly improve discriminability of neural representations and task performance. However, in the visual system, locomotion increases neural responses along with stimulus-decoding accuracy using neural activity at the population level.^5,23–25^ How this relates to behavioral performance is not clear, as performance on visual tasks generally lags behind population-level neural decoding potentials in the visual cortex.^26^ While individual neural responses in the visual system vary in their relationship to locomotion speed,^27,28^ decoding accuracy has often only been tested with a binary classification of states (stationary vs. locomoting). Binary classification of locomotion in some instances indicates that locomotion is beneficial for task performance, while others have found it to be detrimental.^15,29^ In the present study, the probability of both task disengagement and sub-optimal strategic states increased athigher levels of both the continuous pupil diameter and movement indexes, leading to decreased performance at high arousal/movement levels across both auditory and visual modalities. Crucial to this observation is that movement is measured on a continuous scale. In addition, effects on stimulus-decoding accuracy have only been performed in passive contexts and are restricted to the visual cortex. While arousal-dependent cortical decoding capacities may contribute to behavioral performance, other brain regions receiving their signals may have different arousal-dependent dynamics, where heightened arousal may inhibit optimal performance.

Regulation of arousal to maintain ideal levels for decision-making may lead to increased performance. During difficult tasks, external feedback based on optimal neural activity in humans can increase performance and decrease arousal as measured by decreases in pupil diameter and increases in heart rate variability,^30^ consistent with the right half of the Yerkes-Dodson relationship. Internal factors may also regulate arousal. Development during childhood and adolescence leads to self-regulation of arousal, contributing to executive function in humans.^31–33^ This developmental phenomenon is not unique to humans−in mice, projections from the prefrontal cortex (PFC) to the serotonergic dorsal raphe nucleus (DRN) increase through adolescence, and their emergence coincides with increases in persistence during active foraging.^34^ Recent work has shown that humans can gain volitional control of pupil size through training, systematically regulating neural structures related to arousal.^35^ In the present study, in addition to finding an ideal range of pupil diameters for optimal task performance, we report a decrease in trial-to-trial variability of all recorded arousal measures during epochs of optimal performance states and differing optimal pupil diameters based on sensory modality (Figures 4A and 3D). Additionally, the correlation between movement and pupil diameter seen during disengagement is degraded during optimal performance epochs, suggesting a context-dependent selective decoupling of arousal from movement. While we do not present direct evidence of volitional control of arousal in mice, the arousal regulation shown during optimal engagement states opens questions regarding the mechanisms that contribute to this effect. While strategic control of movements may account for some of the arousal changes, further study into the use and development of regulatory mechanisms and contextual control of arousal is warranted.

What is the neural mechanism underlying performance state switching and its arousal-induced regulation? Fluctuations in pupil diameter are correlated with activity in arousal-linked neuromodulatory centers in mice, non-human primates, and humans.^20,36,37^ The PFC is well situated to be an orchestrator of neuromodulatory centers linked to arousal and pupil size. In mice, PFC projections target both serotonergic and GABAergic populations in the DRN, and electrical stimulation of the PFC modulates DRN activity.^38–40^ Direct activation of serotonergic cells in the DRN sustains arousal, slowing pupil constriction.^41^ Similarly, direct stimulation of the noradrenergic locus coeruleus (LC) leads to increases in pupil diameter, and stimulation of PFC projections to inhibitory populations surrounding the LC leads to pupil constriction.^42^ PFC projections can act as dynamic regulators for key arousal-linked neuromodulatory centers, and their coordination could maintain optimal arousal levels during task engagement. Further work is necessary to determine the activity and influence of such projections during various task-engagement states.

### Limitations of the study

The current study utilizes GLM-HMMs of behavior, which assume discrete transitions between performance states. While GLM-HMMs have been shown to fit similar datasets better than models with continuous dynamics,^16^ continuous transitions between performance states may occur. We account for this using a confidence threshold on state posterior probabilities for inclusion within a discrete state. Further model development could account for both discrete and continuous transitions in decision-making dynamics.

When making comparisons of optimal pupil diameter across the auditory and visual tasks, distinct mice were used. This requires comparison of a relative measure across sessions and mice. To account for this, we normalize pupil diameter to the maximum within each session, as detailed in the STAR Methods. For the significance of the comparison to be erroneous under this scheme, auditory-task-performing mice would need to have systematically reached higher arousal levels than visual-performing mice each session, which we assert is implausible, as subjects were handled by the same experimenters and run inter-leaved in time in the same training rigs.

## STAR★METHODS

### RESOURCE AVAILABILITY

#### Lead contact

Further information requests should be directed to and will be fulfilled by the lead contact, Santiago Jaramillo (sjara@uoregon.edu).

#### Materials availability

This study did not generate new unique reagents or mouse lines.

#### Data and code availability

- Neurodata without borders files (.nwb) with all task and physiological measure data have been deposited on DANDI and are publicly available at https://doi.org/10.48324/dandi.000678/0.231004.2146.
- Analysis code is deposited at Zenodo and is publicly available at https://doi.org/10.5281/zenodo.10306018.
- Any additional information required to reanalyze the data reported in this work paper is available from the lead contact upon request.

### EXPERIMENTAL MODEL DETAILS

#### Mice

All procedures were carried out with approval from the University of Oregon Institutional Animal Care and Use Committee. Animals (female and male mice, 8–15 weeks at time of surgery) were of C57BL/6J background purchased from Jackson Laboratory and bred in-house, including wild-type C57BL/6J, transgenic Cre and tTA driver lines (CaMK2-Cre, Jax #005359; CaMK2-tTA, Jax #007004), and fluorescent reporter lines (tetO-GCaMP6, Jax #024742; TIGRE2.0 GCaMP6s Jax #31562). Crosses of drivers and reporters yielded in usable double heterozygous mice (CaMKII (+/−) x GCaMP6s (+/−)). The mice were kept on a reverse light cycle and had *ad-libitum* access to food and water until time of behavioral training. A total of 31 mice were used in this study, with 13 reaching performance criterion for inclusion in final analysis. We did not assess the influence of sex on the results of this study. This is limitation of the study.

### METHOD DETAILS

#### Headplate implantation

All surgical procedures were performed in an aseptic environment with mice under 1–2% isoflurane anesthesia with an oxygen flow rate of 1.5 L/min, and homeothermic maintenance at 36.5°C. Mice were administered systemic analgesia (Meloxicam SR: 6 mg/kg, Buprenorphine SR: 0.5 mg/kg) and a fluid supplement (1 mL lactated ringer’s solution) subcutaneously. Fur was removed across the dorsal and right temporal surfaces of the skull, and the skin was sterilized with povidone/iodine solution followed by isopropyl alcohol three times over. The skin, connective tissue, and part of the right temporalis muscle were removed, and the exposed skull was cleaned. A custom-designed headplate^44^ was affixed to the skull using dental cement (RelyX Unicem Aplicap, 3M), and skin was affixed to the outside edge of the headpost as necessary (Vetbond, 3M). The exposed skull was covered using cyanoacrylate (Slo-zap, Zap), and protected with a silicone elastomer (Kwik-Sil, World Precision Instruments). Mice recovered for three days in an incubator recovery chamber, and lactated ringer’s solution was administered as necessary.

#### Task details

Data collection and stimulus presentation was conducted using custom LabView (National Instruments) scripts. Mice were headfixed on a running wheel and trained on either an auditory or a visual stimulus discrimination task to receive water rewards. Mice made a binary classification of stimuli, and reported their choice by licking one of two reward ports (left and right) spaced 500 μm apart.

For the auditory task, stimuli consisted of tone clouds with three concurrent streams of tones, where each tone lasted 30 ms and had a frequency selected between 5 and 40 kHz. Tone clouds were differentiated by selecting a varied proportion of tone frequencies in either the bottom (5–10 kHz) or top (20–40 kHz) octave of the range. The remaining tones were randomly distributed across the rest of the frequency range. Auditory stimulus values are defined based on the proportion of tones in the right-target octave (e.g., high frequency) minus the proportion of tones in the left-target octave (e.g., low frequency), such that −1 represents a tone cloud with all tones in the left-target octave, −1 represents a tone cloud with all right-target octave tones, and 0 represents a stimulus with an equal number of tones in each target octave. Tones were calibrated to 60 dB SPL and waveforms were generated (NI PXI-4461, National Instruments) at 200 kHz sampling rate, conditioned (ED1, Tucker Davis Technologies), and transduced by electrostatic speakers (ES1, Tucker Davis Technologies).

For the visual task, stimuli consisted of drifting Gabor patches displayed on an LED screen with a refresh rate of 30 Hz. Stimuli had a constant mean luminance, matched to the static gray background displayed between stimuli. Each Gabor had 0.08 cycles per degree of visual field and drifted at 1.5 cycles per second. Gabor angles were between 0 and 90°, and differentiated by being closer to a horizontal (e.g., 0, 18, or 36°) or vertical (e.g., 90, 72, or 54°) orientation. Visual stimulus values were defined between 0 and 1 by the normalized difference of their angle from 45°, and signed in accordance with their directional representation (negative if left, positive if right).

Stimuli (high/low tone cloud or horizontal/vertical gabor) were randomly assigned to left or right identities for each subject at the beginning of training. In both tasks stimuli lasted 1.2 s and were presented with an inter trial interval (ITI) of 5 ± 2 s, which was reset to licking prior to stimulus presentation. Licking of reward ports was electrically monitored at 1 kHz (USB-6008, National Instruments). If mice responded to stimuli by licking the appropriate reward port during the stimulus or within 1 s of the stimulus offset, the trial was considered a hit and a 3 μL reward was delivered through the port by a syringe pump (NE-500, Pump Systems Inc.). If the incorrect port was licked first during the stimulus or response period the trial was considered an error. If no response was made the trial was considered to have no response.

#### Training and inclusion criteria

After postoperative recovery, mice were weighed for three days to establish a baseline weight before beginning a water regulation protocol. After 3–5 days, mice had reached a steady weight on water regulation and began behavioral training. Mice were habituated to human handling, head fixation, locomotion on the wheel and collecting water from the reward ports. Reward port position was adjusted by a manual xyz manipulator. To ensure consistent lick spout positioning, a 3D printed jig was made for each mouse and used to align reward ports at the beginning of each training session.

Behavioral training was conducted using the following stages and progression criteria:

Stage 1 - To encourage licking and associate left and right ports with the appropriate directional stimuli, the left or right port was randomly ‘armed,’ so a lick would prompt appropriate (value −1 if left, 1 if right) stimulus presentation, and water reward delivery. The stimulus and reward were spontaneously delivered after 20 s if the subject did not lick the armed port. The ITI was set to 2 s to promote consistent licking. After 75 such trials in a session, stimuli were presented without a reward, and a reward was delivered if the subject licked the correct port within the response period. If subjects achieved 1500 total licks to each port, and >150 hits in a session, they progressed to stage 2 in their next training session.

Stage 2 - To promote licking only following stimulus presentation, the ITI was extended to 5 ± 2 s, and was reset following aberrant licking for all following stages. Only the easiest stimulus values were used (−1 or 1), and a reward was delivered following any lick to the correct port during the response period. If subjects failed to respond to 10 consecutive trials, a free reward was delivered with the stimulus. If subjects achieved >50% hit rate on both ports, with no less than 30% of licks to each port and >150 hits, they progressed to stage 3.

Stage 3 - To refine choice behavior, a reward was only delivered if the first lick following stimulus presentation was to the correct reward port. To prevent mice from only responding at one reward port, probabilistic bias correction was added, so that more target stimuli of one direction would be presented if that port was being neglected. If subjects achieved >60% hit rate on both ports and >150 hits, with no less than 33% of hits at each port they progressed to stage 4.

Stage 4 - More difficult stimuli were introduced, and probabilistic bias correction was reduced. If subjects achieved >60% hit rate on both ports and >200 hits, with no less than 33% of hits at each port, they progressed to stage 5.

Stage 5 - Bias correction was removed and three difficulties of stimuli are presented per direction. A subset of mice were advanced to stage 6 after at least 10 consecutive stage 5 training sessions.

Stage 6 - Half of the stimuli were of the un-trained sensory modality, presented as distractors. There was no discernible change in task performance, and response rates to the distractors were <10%.

If subjects failed to meet the advancement criteria of a lower training stage for consecutive sessions, they were returned to that training stage. For further analysis we included sessions from stages 5 and 6 with at least 100 rewards delivered, balanced such that no less than 20% of rewards were delivered in each of the left and right ports. Using our 5-fold cross-validation method for model selection (see below) on synthetically generated data required 8–10 sessions to reliably recover the ground truth parameters of the model. Therefore, a minimum of 10 sessions per subject was required for inclusion in further analysis. 13 of 31 mice met inclusion criteria, with a total of 381 of their 500 stage 5 and 6 training sessions included for further analysis. Mice that reached proficiency took 21 ± 8 training sessions to reach stage 5.

#### Modeling task performance

To test the hypothesis that animals switch between discrete decision-making states within single sessions, we developed a hidden Markov model with multinomial Generalized Linear Model observations (GLM-HMM) using a modified version of the SSM python package.^43^ The multinomial GLM observation, parameterized as,

$$\mathrm{Pr}\left(y_{t}=c|z_{t}=k,u_{t},w_{c}^{\left(k\right)},b_{c}^{\left(k\right)}\right)=\frac{\exp\left[w_{c}u_{t}+b_{c}^{\left(k\right)}\right]}{\sum_{c^{\prime }}\exp\left[w_{c^{\prime }}u_{t}+b_{c\prime }^{\left(k\right)}\right]}$$

is the set of three psychometric curves representing the probability of choosing actions c = Left, Right, No response in each trial t, given stimulus and the hidden state. Each hidden state represents a different performance state, including one optimal, and several sub-optimal or disengaged states. A model with K hidden states is described by the following parameters: a KxK transition probability matrix, representing the probability of switching between different states at each trial t ; a K-dimensional vector representing the initial state probabilities; and the observation parameters comprising weights and biases for each of three multinomial categories c=L, R, Nr, the latter corresponding to three choices Left, Right, and No response, available to the subject in each trial. We fit a multinomial GLM-HMM to trials from individual subjects using the Expectation Maximization (EM) algorithm to maximize the log-posterior and obtain the optimized parameters.

Model selection for the number of states was performed using 5-fold cross-validation across sessions of an individual subject. When fitting data generated synthetically with GLM-HMMs, these methods consistently recover the ground truth number of states used to generate the data. Models with up to seven states were fit to the concatenated trials of the training set, and the log-posterior of the test set was estimated (normalized by the number of trials per test set). Because the EM may lead to local maxima of the log-posterior, for each choice of number of states, the EM algorithm was performed 10 times starting from random initial conditions. We performed model selection in two alternative ways: either by maximum likelihood estimation (MLE); or by maximum a posteriori (MAP, including a Gaussian prior on the weights with mean zero and variance equals to 2, and Dirichlet prior on transition probabilities with alpha = 2, see ref. 16 for details on the procedure). The best number of states was chosen as the maximum of the plateau of the test MLE or test MAP log likelihoods. We then fit a single model to the time series of the observations and inputs concatenating all sessions from a subject, optimizing the model parameters using MLE.

For all further analysis we set an 80% state probability criterion for inclusion of a trial within a performance state and consider all other trials as in an indeterminate state. Performance states from the final models were distinct and clearly interpretable, and classified as one of six stereotypic states: optimal (responses congruent with stimulus identity), left bias (leftward licks regardless of stimulus), avoid right (left lick to left-target, no response to right-target stimuli), right bias (rightward licks regardless of stimulus), avoid left (right lick to right-target, no response to left-target stimuli), or disengaged (no response to any stimuli).

The choice prediction accuracy measure was calculated using 5-fold cross validated models. For each test-set trial the GLM-HMM state was inferred using the posterior probabilities calculated from the preceding trials and the state transition matrix. The weights of the inferred state were then used to predict the choice, which is compared to the empirical data to determine model prediction accuracy.

#### Recording arousal measures

All data collection was conducted using custom LabView scripts. While headfixed, subjects were free to locomote on top of a cylindrical wheel with a 15 cm diameter and 20 cm width. The axle of the wheel was connected to a rotary encoder (Model 15T/H, Encoder Products Company), which was used to calculate locomotion speed.

A CMOS camera (Teledyne G3-GM11-M2020, or Basler ace acA780–75gm) with an affixed lens (TEC-M55MPW, or Navitar NMV-50M23) and infrared (IR) filter (MIDOPT BN810–46, or Thorlabs FGL780) was pointed at the face of the subject. The face was illuminated with an IR LED (Digi-Key TSHG8200, 830 nm), and with a white LED (RadioShack 5 mm 276–0017). Prior to each recording session, illumination conditions were optimized with live feedback and online pupil fitting. IR lighting was adjusted to provide even illumination of the face and eye without shadows, and the ambient light intensity adjusted so the pupil would have a large dynamic range, while not being obscured by the eyelid when maximally dilated. Images of the face were acquired at 30 Hz throughout task performance, and time stamps for each frame were saved at time of acquisition.

Online pupil diameter and face motion energy estimates were made using LabView software, and post-hoc analysis was done using custom python scripts. Face motion energy was calculated within a rectangular ROI anterior to the eye (Figure 3A). The absolute value of the frame-to-frame change in pixel intensity was averaged within the ROI and normalized to the maximum within a session. To calculate pupil diameter post-hoc, first an ROI around the eye was selected and the area displayed at regular intervals throughout the session, so an appropriate binarization threshold could be manually selected. The contour of the pupil was extracted for each frame, and the long axis of a fit ellipse was recorded as the pupil diameter. The quality of the pupil values was verified for each session by viewing the dynamics of the full session traces for online and post hoc calculations along with images of the pupil fit at the time of the maximum and minimum calculated values within a session. This allowed rapid identification and exclusion of anomalous frames. If dropped frames resulted in gaps less than 200 ms, pupil data were interpolated. To allow comparison across sessions and mice, all pupil diameters were normalized to the maximum within a training session. This method assumes similar maximal levels of arousal are reached each session, but accounts for differences in anatomy and illumination.

All measures were smoothed with second order Savitzky Golay filters with 200 ms (face energy and locomotion speed) or 500 ms (pupil diameter) windows and upsampled to 1 kHz. In order to determine the influence of tonic arousal and movement on performance, the value of each measure at the time point sampled immediately before stimulus presentation began was used for further analysis. In addition to the raw values, the standard deviation and coefficient of variation of the measures’ values over the past 10 trials were calculated for each trial.

The measures of face motion energy and locomotion speed individually account for qualitatively distinct magnitudes of movement in mice during head fixation. Face motion energy effectively captures change in low-medium ranges of movement while the subject is stationary (not walking), and locomotion speed effectively differentiates medium-high output movements. In order to create a single value representative of a broad range of movement, the values of face motion energy and locomotion speed were z-scored and summed to create a single continuous movement index. The same was done for the past 10 trial standard deviation values.

#### Determining optimal pupil diameters

Probability of a performance state was calculated in relation to each arousal measure by binning trials by measure values and determining the proportion of trials in each performance state. In order to determine the optimal pupil diameter for each subject, polynomial functions were fit to the optimal state occupancy probability across pupil diameters. Polynomial degree for a fit was selected by determining the elbow of the test set r^2^ increase function with 5-fold cross validation. A final polynomial was fit to all data, and the optimal pupil diameter was determined for each subject as the maximum of the fit curve within the range of the true data. The unique optimal pupil value for each subject was used to align pupil values for visualization, and to calculate the absolute difference from the optimal pupil diameter for each trial.

#### Performance state classification

We used cross-validated classifiers (support vector machines with a radial basis function kernel) to discriminate trials from the optimal performance states against trials of each of the disengaged and sub-optimal states (binary classifications) using six features: the 3 recorded behavioral measures and their variability over the 10-past-trials. Classification was performed on a randomly selected equal number of trials of each performance state using 5-fold cross-validation. Accuracy is reported as the mean accuracy of test set classification across five such folds. To test the significance of the reported accuracies, we z-scored the empirical accuracy against 1000 classifiers trained on surrogate data obtained by randomly permuting class labels.^45^ To determine the contribution of features to classification accuracy, we compared the performance of the classifiers trained on the empirical data to those trained on surrogate data obtained by shuffling the labels for a subset of features across trials within the training set.

### QUANTIFICATION AND STATISTICAL ANALYSIS

Statistical analysis was performed using SciPy1.5.2 (Python). Sample sizes were not predetermined with statistical methods but are similar to other studies in the field. Statistical details can be found in each figure legend and associated results sections. Data and statistics are reported as the mean ± standard error of the mean unless otherwise noted. Boxplots represent the first and third quartiles, with the median represented as a bar., and whiskers representing 1.5 times the interquartile range. Two-tailed Wilcoxon signed rank (paired) or rank sum (unpaired) statistical tests were used to avoid assumptions regarding the normality of data distribution. All comparisons were made over mice unless otherwise noted. Individual data points are shown when possible.

## Supplementary Material

1

## Figures and Tables

**Table:** KEY RESOURCES TABLE

REAGENT or RESOURCE	SOURCE	IDENTIFIER

**Deposited data**		

Mouse behavioral data	This paper, DANDI archive	https://doi.org/10.48324/dandi.000678/0.231004.2146

**Experimental models: Organisms/strains**		

Mouse: C57BL/J6	The Jackson Laboratory	Stock number #000664
Mouse: CaMKII-Cre	The Jackson Laboratory	Stock number #005359
Mouse: CaMKII-tTA	The Jackson Laboratory	Stock number #007004
Mouse: tetO-GCaMP6s	The Jackson Laboratory	Stock number #024742
Mouse: Ai162D-GCaMP6s	The Jackson Laboratory	Stock number #31562

**Software and algorithms**		

LabView 2016	National Instruments	RRID: SCR_014325
Python	Python Software Foundation	RRID:SCR_008394
Python original analysis code	This paper, Zenodo	https://doi.org/10.5281/zenodo.10306018
Python uobrainflex package	This paper, Github	https://github.com/sjara/uobrainflex
Python SSM package	(Underman et al. 2 0 20),^43^ Github	https://github.com/lindermanlab/ssm
SciPy1.5.2	SciPy	https://docs.scipy.org/doc/scipy-1.5.2/reference/

**Other**		

PXI Sound and Vibration Module	National Instruments	NI PXI-4461
Speaker driver	Tucker Davis Technologies	ED1
Electrostatic speaker	Tucker Davis Technologies	ES1
White LED	RadioShack	5 mm 276–0017
Infrared (IR) LED	Digi-Key	TSHG8200, 830 nm
CMOS camera	Teledyne	G3-GM11-M2020
Affixed lens	Computar	TEC-M55MPW
Infrared (IR) filter	Thorlabs	FGL780
CMOS camera	Basler	ace acA780–75gm
Affixed lens	Navitar	NMV-50M23
Infrared (IR) filter	MIDOPT	BN810–46
Rotary encoder	Encoder Products Company	Model 15T/H
Reward pump	Pump Systems Inc	NE-500
Multifunction I/O device	National Instruments	USB-6008
Headplate	(Vickers and McCormick, 2023)^44^	A1/V1/M2 headplate
Meloxicam SR	Wedgewood Pharmacy	Meloxicam SR (2 mg/mL)
Buprenorphine SR	Wedgewood Pharmacy	Buprenorphine SR (0.5 mg/mL)
Isoflurane	Vetone	Fluriso
Cyanoacrylate	Pacer technology (ZAP)	SloZap (thick)
Dental Cement	3M	RelyX Unicem Aplicap
Tissue adhesive	3M	Vetbond
Silicone elastomer	World Precision Instruments	Kwik-Sil

## Highlights

- Mice transition between distinct performance states during auditory and visual tasks
- Epochs of optimal performance are selectively maintained for extended durations
- Correlations between movement and pupil diameter are disrupted during optimal state
- Moderate levels (and reduced variability) of pupil and movement predict optimal state
