# Supplementary material for: Decision-making dynamics are predicted by arousal and uninstructed movements
Source: Cell Rep. Author manuscript; Available in PMC 2024 Apr 14. (PMC11016285; doi:10.1016/j.celrep.2024.113709)
Supplement: 1 [file NIHMS1970871-supplement-1.pdf]

Cell Reports, Volume *43*

Supplemental information

Decision-making dynamics are predicted
by arousal and uninstructed movements

Daniel Hulsey, Kevin Zumwalt, Luca Mazzucato, David A. McCormick, and Santiago Jaramillo

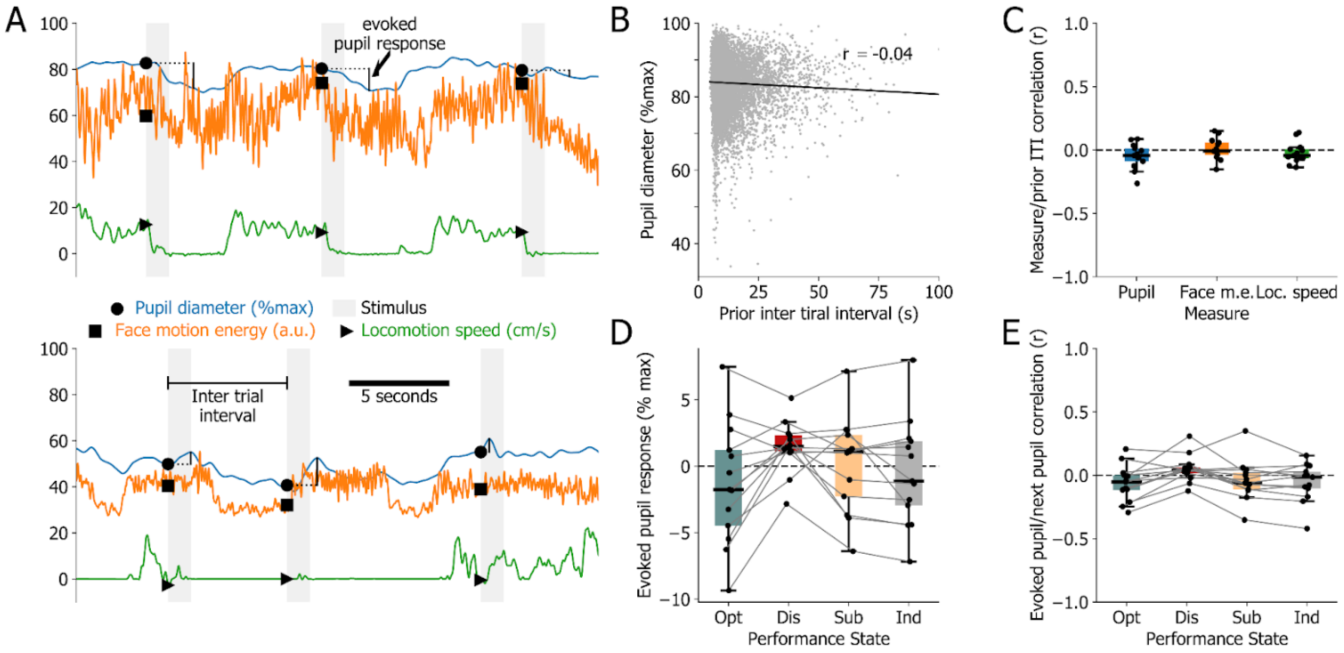

Figure S1. Inter trial interval of task is sufficient in duration to prevent evoked physiological responses from substantively influencing subsequent trials. Related to figure 1 **(A)** Two examples of physiological measures recorded during task performance. Fluctuations in movement measures generally precede similar fluctuations in pupil diameter. In the top example the mouse stops locomoting upon stimulus presentation, resulting in a decrease in pupil diameter in response to the stimulus. In the bottom example the mouse is not locomoting prior to stimulus presentation, and there is an increase in pupil diameter following the stimulus. Note that multiple phases of movement and pupil fluctuations occur prior to the beginning of the subsequent trial. **(B)** The correlation between pupil diameter at the start of each trial and prior inter trial interval is very weak for this example subject. **(C)** Correlations between all measures at the start of a trial and prior inter trial intervals are weak and centered around zero across mice. **(D)** Average evoked pupil responses of mice are not significantly different across performance states (Kruskal-Wallis test, $p = 0.54$). **(E)** Correlations between evoked pupil response and pupil diameter at the beginning of the subsequent trial are very weak and centered around zero across mice. All boxes extend between the lower and upper quartiles, with a line at the median, and whisker extending to the last data point within 1.5 times the inter quartile range.

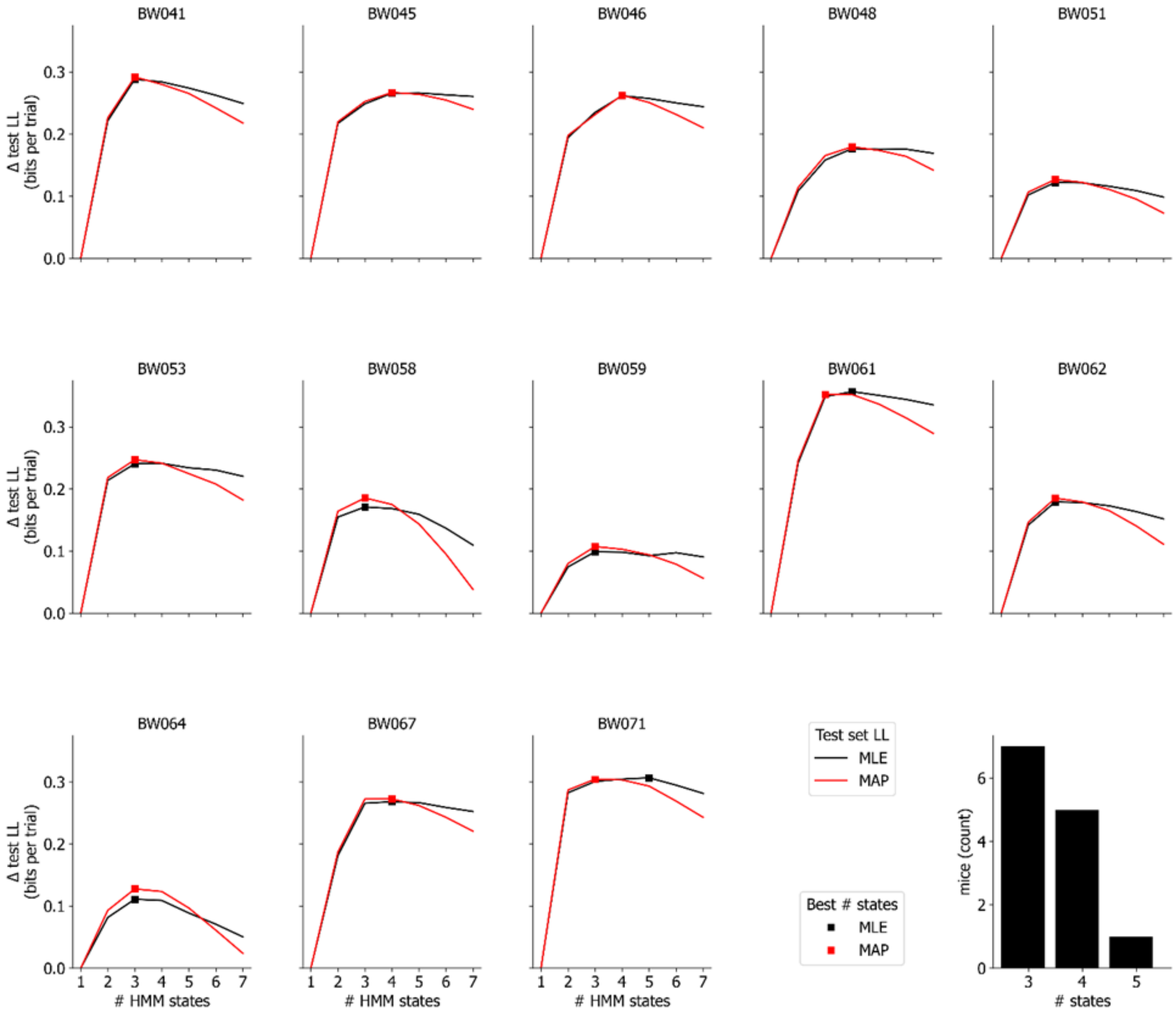

State	BW041	BW045	BW046	BW048	BW051	BW053	BW058	BW059	BW061	BW062	BW064	BW067	BW071
Optimal	x	x	x	x	x	x	x	x	x	x	x	x	x
Disengaged	x	x	x	x	x	x	x	x	x	x	x	x	x
Bias left			x	x		x			x			x	x
Avoid right		x			x		x				x		
Bias right		x	x						x			x	x
Avoid left	x			x				x		x			x

Figure S2. Selection of number of HMM states. Related to Figure 1. State selection was performed using five-fold cross validation of test set log likelihood (LL), training models on 80% of sessions, and testing the fit on the remaining 20%. Both MLE and MAP methods were used (see materials and methods), with ten initializations per state and model type, and the maximum LL acquired was kept. The plateau of the average LL across the five folds was considered the appropriate number of states to use for final model fitting. In case of discrepancies between MLE and MAP states, the larger number of states was used.

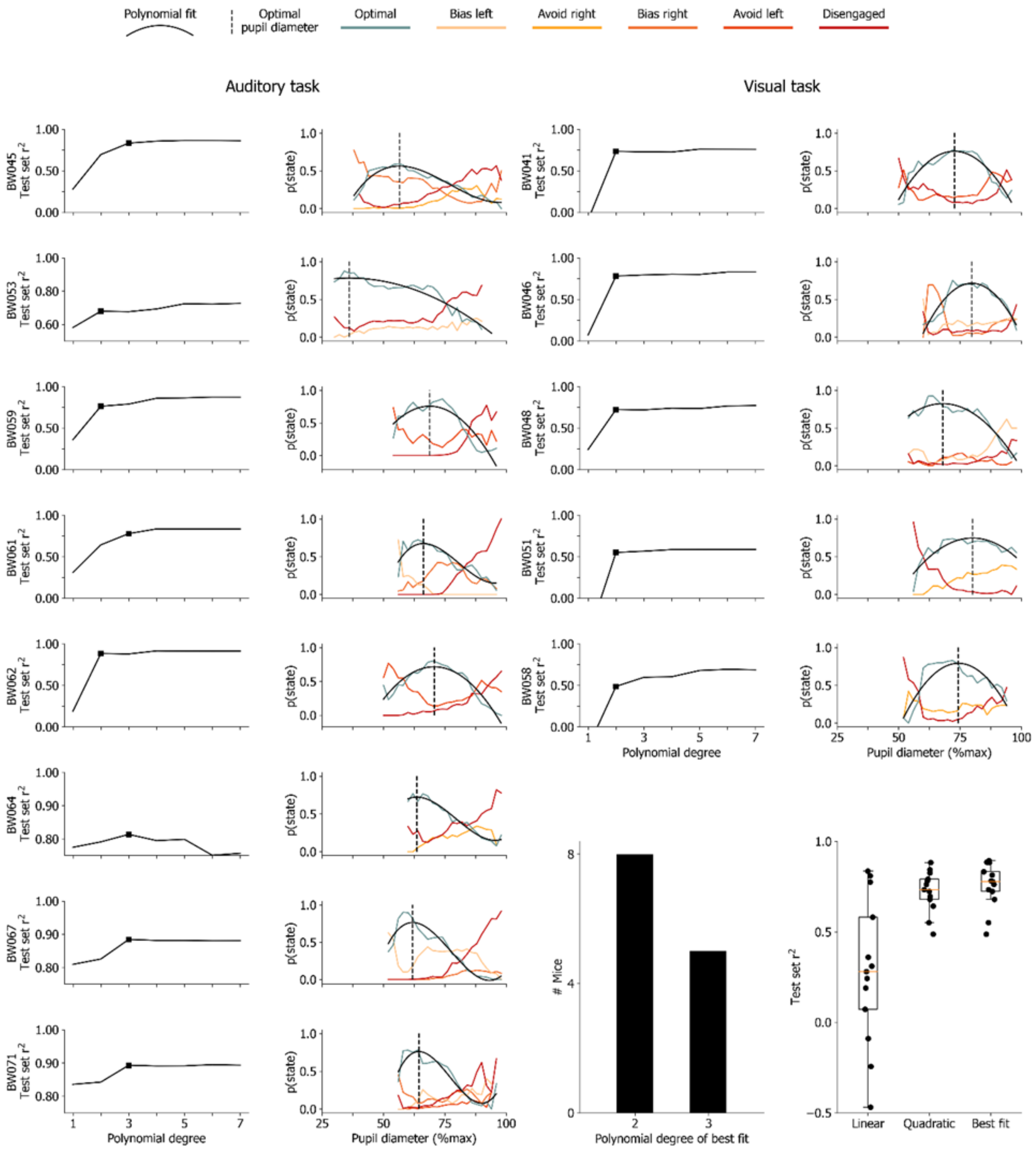

Figure S3. Optimal pupil diameter calculated for individual subjects. Related to Figure 3. Polynomial functions were fit to the optimal state occupancy probability data for each individual mouse. Polynomial degree was selected using 5 fold cross-validation, and the elbow of the function was used to fit a final curve to all data. For the majority of mice, a quadratic function was sufficient to explain most of the variability. A cubic function was better fit in individuals with long tails to the inverted-U relationship. The optimal pupil diameter for each subject was defined as the maximum along the fit curve within the range of the true pupil data. boxes extends between the lower and upper quartiles, with a line at the median, and whiskers extend to the last data point within 1.5 times the inter quartile range.

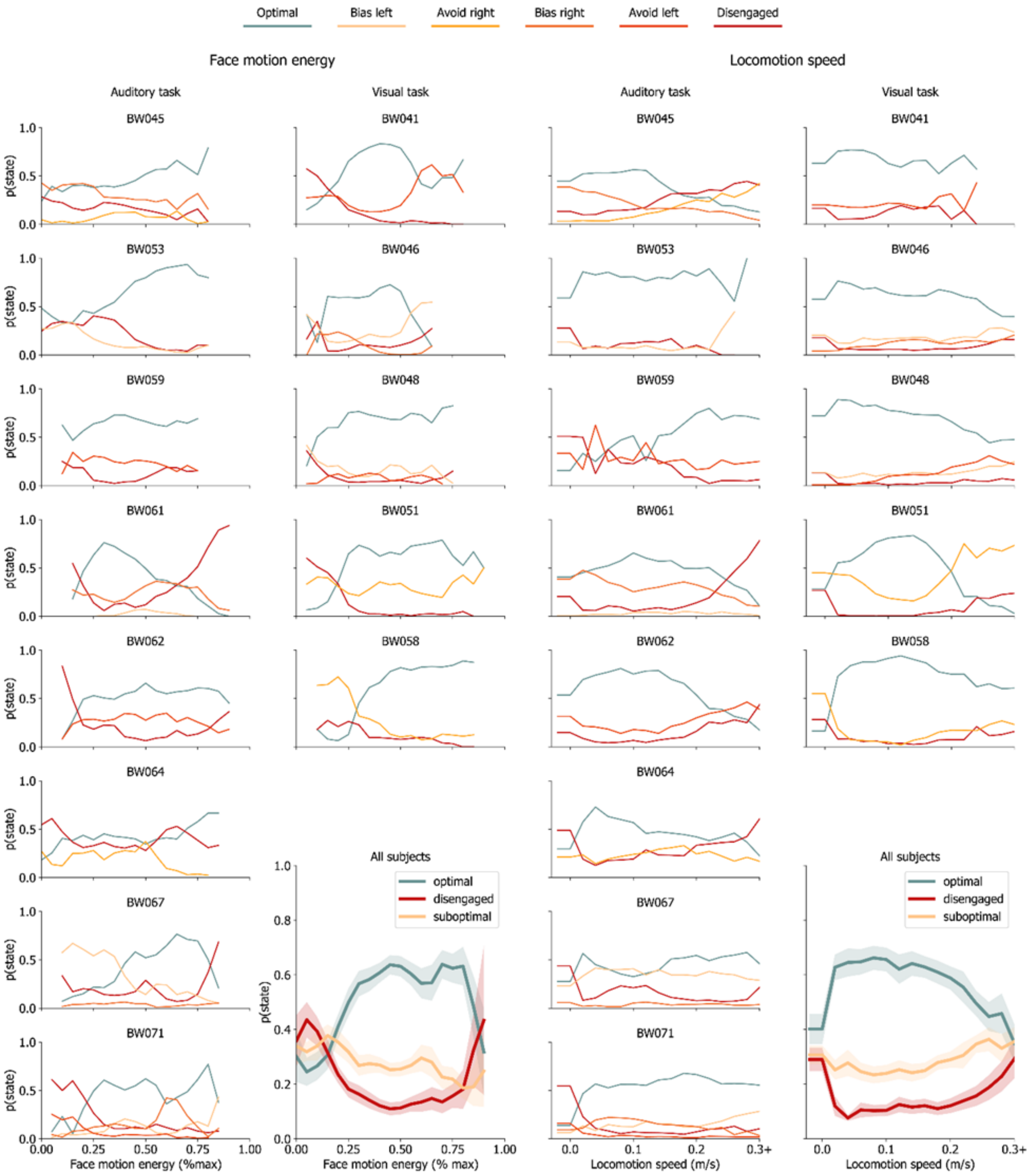

Figure S4. Relationship between optimal state occupancy and individual movement measures are variable. Related to Figure 3. Individual mice have inverted-U relationships between optimal state occupancy and face motion energy or locomotion speed. However, the relationship is inconsistent, and not found in all subjects. Across subjects, face motion energy forms a largely monotonic relationship, while locomotion speed has a moderate inverted-U relationship. all subject plots represent the mean ± SEM across mice (N = 13).

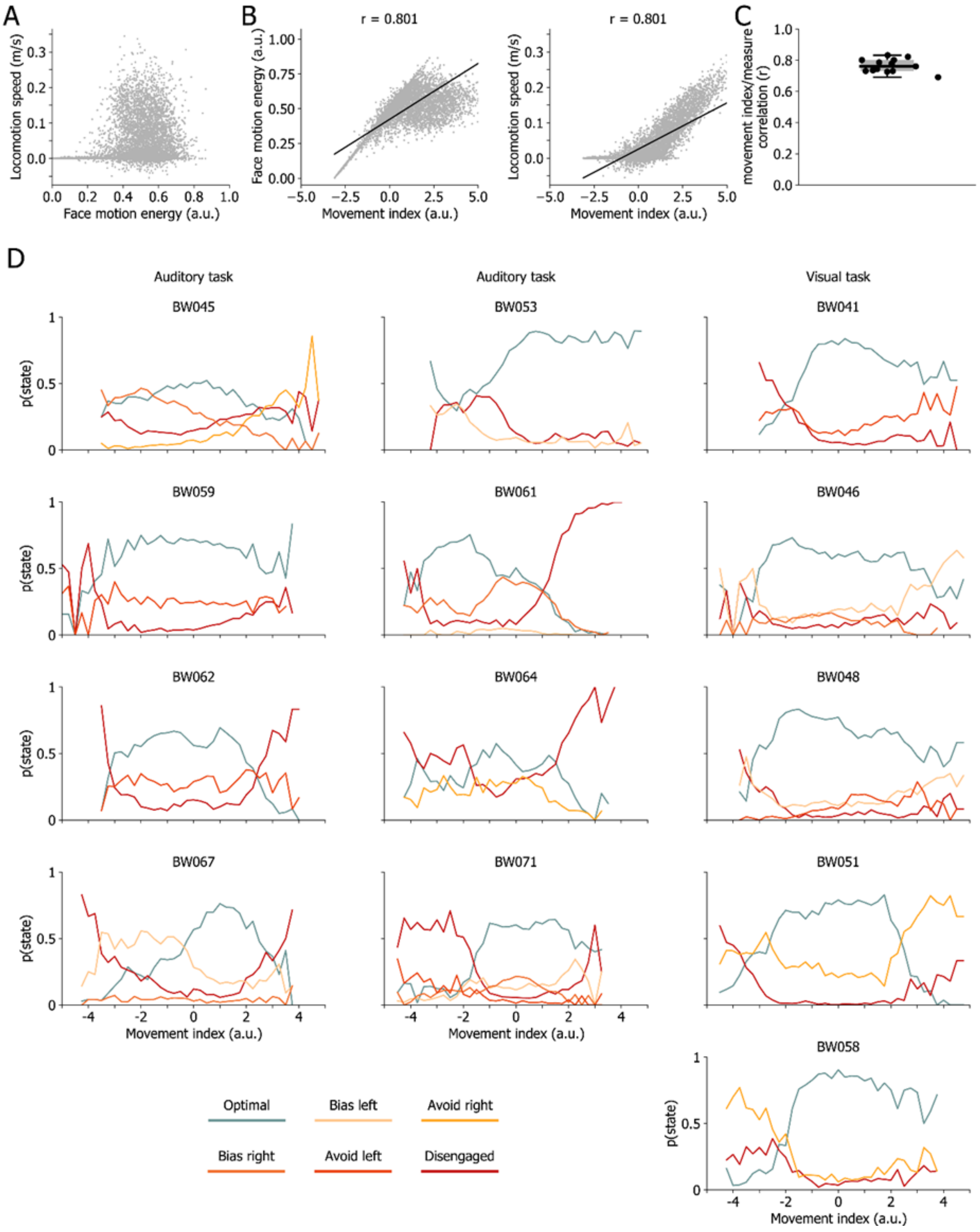

Figure S5. Movement index creates a single measure incorporating locomotion and face motion energy. Related to Figures 3 and 4**. (A)** Scatter plot of coincident face motion energy and locomotion speeds for all trials of an example subject. Small face motion energy levels only occur during periods of no locomotion. **(B)** Calculation of movement index yields identical correlations to both face motion energy and locomotion speed values. r values are of Pearson correlation coefficients. **(C)** Motion index for each mouse is highly correlated to their individual movement measures. **(D)** Inverted-U relationships between optimal state occupancy and movement index is consistent across mice.

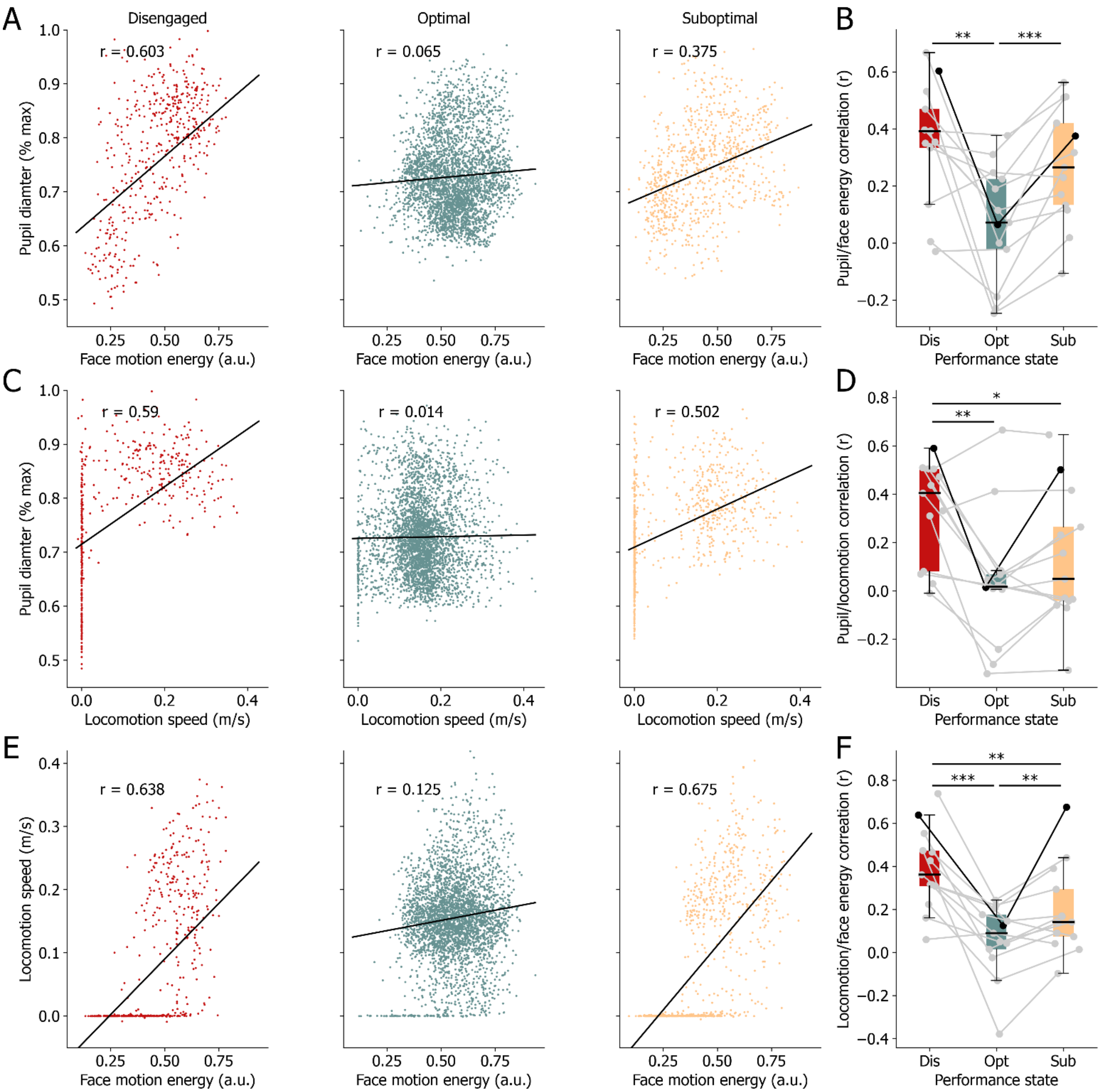

Figure S6. Correlations between individual movement and arousal measures are disrupted during optimal state occupancy. Related to Figure 4. **(A)** Correlations between pupil diameter and face motion energy across GLM-HMM performance states for an example mouse. **(B)** Across mice there is a significant decrease in the correlation between pupil diameter and face motion energy during optimal state occupancy. **(C, D)** As in (A) and (B), but for correlations between pupil diameter and locomotion speed. **(E, F)** As in (A) and (B), but for correlations between locomotion speed and face motion energy. All r values are of Pearson correlation coefficients. All boxes extend between the lower and upper quartiles, with a line at the median, and whisker extending to the last data point within 1.5 times the inter quartile range. * indicates $p < 0.05$, ** indicates $p < 0.01$, *** indicates $p < 0.001$ using Wilcoxon signed rank tests across subjects (N = 13).

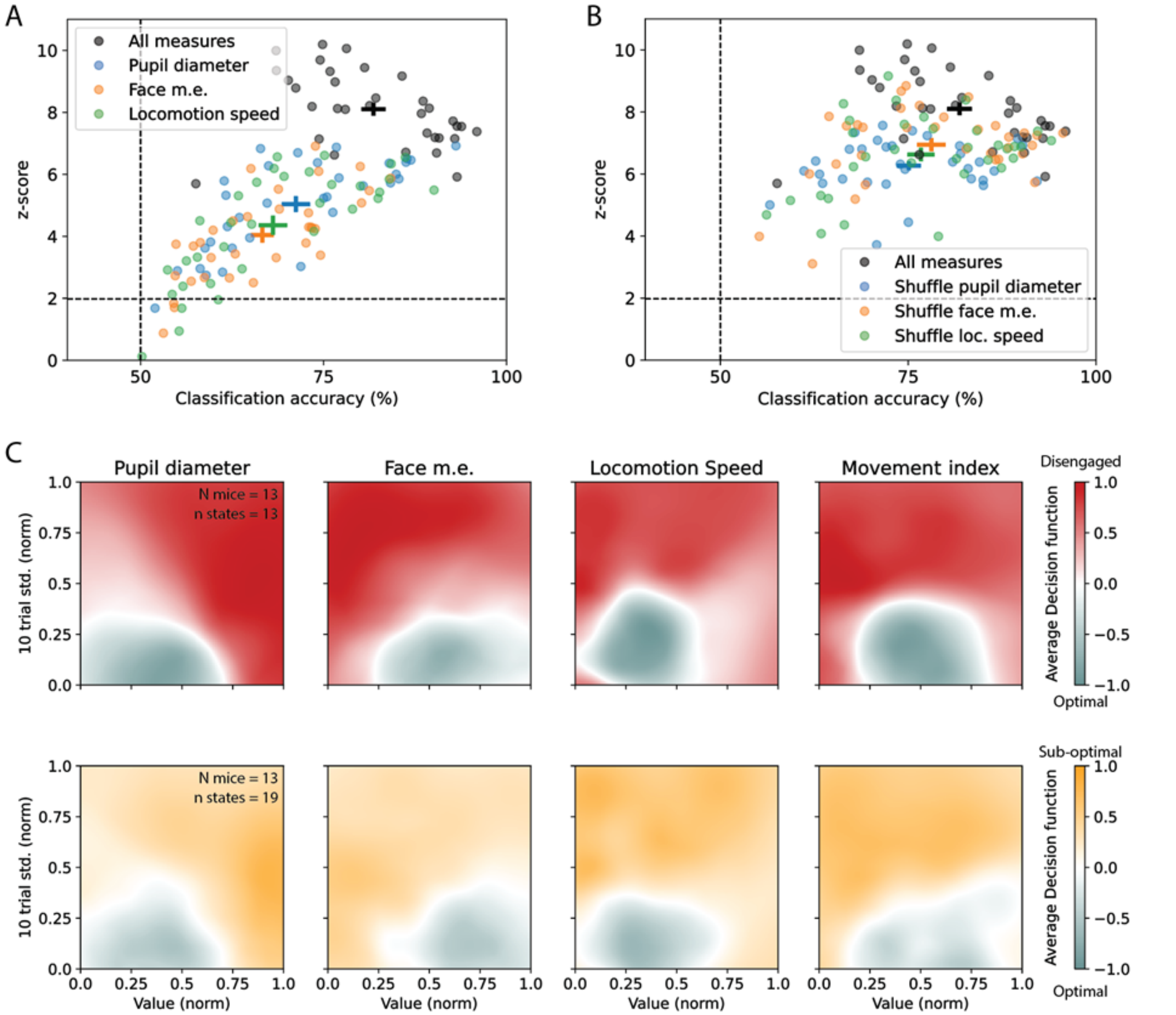

Figure S7. Arousal measure contributions to performance state classification. Related to figure 5. **(A)** State classification using raw movement measures shows that pupil diameter is the best single measure used. Data points represent average performance state classification accuracy across test sets and z-scores against test set accuracy of classifiers trained on state-shuffled data. Crosshairs represent mean ± SEM across all state classifications (n = 32; 13 optimal vs disengaged, 19 optimal vs suboptimal). **(B)** As in (A) but shuffling the value and standard deviation of individual measures indicates their unique contributions. Pupil diameter contributes significantly more to decoding than face motion energy (p = 0.004, Wilcoxon signed rank test), but only slightly more than locomotion speed (p = 0.14, Wilcoxon signed rank test). **(C)** Average decision functions across all subjects for disengaged (top) and sub-optimal states (bottom). Note that the low variability consistently predicts optimal state, along with intermediate levels of pupil, locomotion, and overall movement.

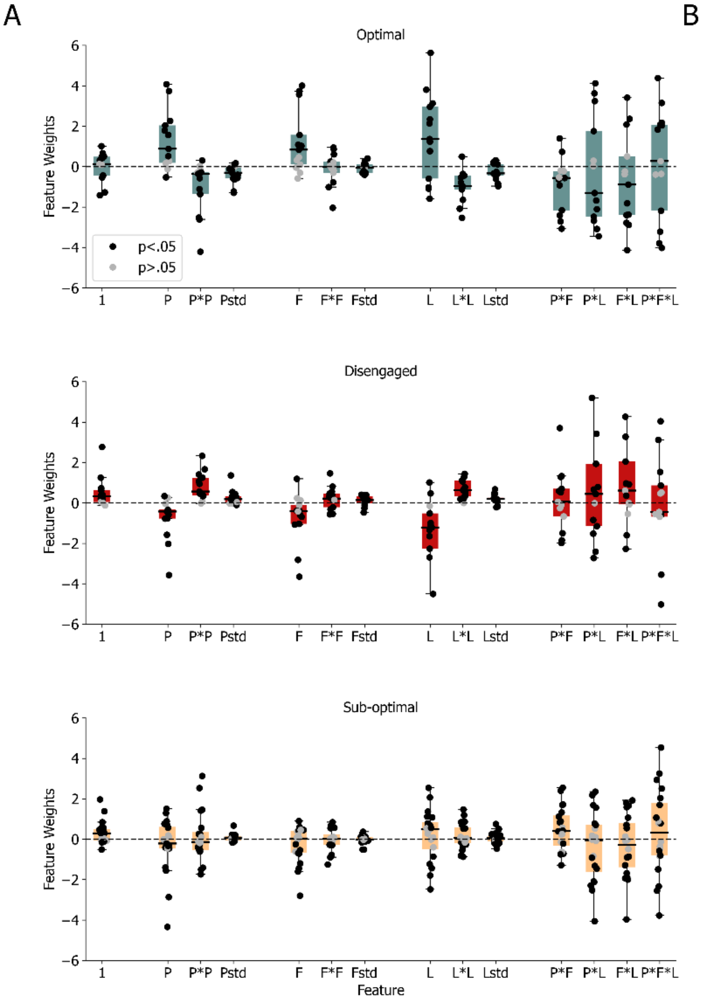

Figure S8. Weights from multi-factor linear regressions corroborate individual measure-state relationships. Related to figures 3 and 5. **(A)** Weights of linear regressions fit to the trial-wise GLM-HMM performance state probabilities using features of pupil diameter (P), face motion energy (F), locomotion speed (L), their past 10-trial variability, quadratic, and interaction terms. Pupil and locomotion speed measures consistently have weights indicative of an inverted-U (negative weights on quadratic terms) when fitting optimal state probabilities, and weights consistent with a U-shaped relationship when fitting disengaged state probabilities. **(B)** Same as in (A) but using the movement index instead of individual measures of face motion energy and locomotion speed. The movement-pupil interaction term is the only feature consistently weighted across subjects for fits to sub-optimal states. All boxes extend between the lower and upper quartiles, with a line at the median, and whisker extending to the last data point within 1.5 times the inter quartile range.
